# Editorials

**Published:** 1891-06

**Authors:** 


### HOMŒOPATHIC PHYSICIAN,

A MONTHLY JOURNAL OF

HOMŒOPATHIC MATERIA MEDICA AND CLINICAL MEDICINE.

" If our school ever gives up the strict inductive method of Hahnemann, we are lost, and deserve only to be mentioned as a caricature in the history of medicine."—CONSTANTINE HERING.

Vol. XI. JUNE, 1891. No. 6.

### EDITORIALS.

SUPERSTITIONS.—In this enlightened age, as we are wont to term it, superstition is usually looked upon as an attribute of the ignorant only, or of the uncivilized. It requires but slight observation in the sick-room to see that many who boast of intelligence are under its influence.

A lack of what is supposed to be possessed by many—common sense—is found very often at the bedside. There are to be found some of the wildest ideas regarding the proper care of the sick.

There is no greater fallacy in existence than what is well termed the " fresh-air superstition," more particularly regarding night air.

If ordinary " horse sense " be applied to the subject we should understand that, no matter what be the nature of the trouble, fresh air is as of much importance in the treatment of the sick as the best selected medicine. Without it we cannot hope for much improvement in any case.

There is no doubt on our part that the majority of cases of sickness are due to the want of pure air in our houses—the microbe theory to the contrary notwithstanding.

If this superstition were confined to the laity only it would be all the better for the sick, for then the practical physician could do much to allay it; but unfortunately there are many who are known as physicians who are equally superstitious. There is no greater than the night-air superstition. What! have a sick person breathe the night air? How under heaven is he to breathe if he does not breathe night air? The bugbear malaria is always offered as an unanswerable argument. As if there could be found worse malaria than in an unaired room! To healthy nostrils, and equally healthy lungs, nothing is more nauseating than an occupied room from which the air has been excluded during the night—and nothing is more unhealthy.

Hygeia, brought into such contact is outraged, insulted, and endeavors to rid herself of the poison by creating nausea and even vomiting.

Tuberculosis, pneumonia, and all contagious diseases are more often due to living without sufficient fresh air and light, plus heredity and allopathy, than to any other one cause.

Go into a house where there is malignant diphtheria, and there you will find the odor peculiar to all such places; not only the characteristic odor of the disease, but also that which comes from a want of air and light. And there, usually, you will find so-called disinfectants. There is no disinfectant in existence comparable to fresh air, and none so efficacious.

Open windows and a breeze through the room are of more value than all the so-called deodorizers and disinfectants known.

The following, from Dr. Felix Oswald, should be spread broadcast: "The influence of anti-naturalism is most strikingly illustrated in our dread of fresh air. The air of the out-door world, of the woods and hills, is, *par excellence*, a product of Nature—of wild, free, and untameable Nature—and therefore the presumptive sources of innumerable evils.

"Cold air is generally the scapegoat of all sinners against Nature. When the knee-joints of the young debauchee begin to weaken, he suspects that he has 'taken cold.' If an old glutton has a cramp in his stomach, he ascribes it to an incautious exposure on coming home from a late supper. Toothache is supposed to result from draughts; croup, neuralgia, mumps, etc., from raw March winds. When children have to be forced to sleep in unventilated bed-rooms till their lungs putrefy with their own exhalations, the mother reproaches herself with the most sensible thing she has been doing for the last hundred nights—opening 'the windows last August when the air was so stiflingly hot.'

"The old dyspeptic, with his cupboard full of patent nostrums, can honestly acquit himself of having yielded to any natural impulse; after sweltering all summer behind hermetically closed windows, wearing flannel in the dog-days; abstaining from cold water when his stomach craved it; swallowing drugs till his appetite has given way to chronic nausea, his conscience bears witness that he has done what he could to suppress the original depravity of Nature; only once the enemy got a chance at him: in rummaging his garret for a warming pan he stood for half a minute before a broken window—to that half minute, accordingly, he attributes his rheumatism.

"For catarrh there is a stereotyped explanation: 'caught cold.' That settles it. The invalid is quite sure that her cough came on an hour after returning from that sleigh-ride. She felt a pain in the chest the moment her brother opened that window. There is no doubt of it—it is all the night-air's fault.

"The truth is that cold air often reveals the existence of a disease. It initiates the reconstructive process, and thus apparently the disease itself; but there is a wide difference between a proximate and an original cause. A man can be too *tired* to sleep, and too *weak* to be sick. The vital energy of a person breathing the stagnant air of an unventilated stove-room is often inadequate to the task of undertaking a restorative process—though the respiratory organs, clogged with phlegm and all kinds of impurities, may be sadly in need of relief. But during a sleigh-ride, or a few hours' sleep before a window left open by accident, the bracing influence of the fresh air revives the drooping vitality, and Nature avails herself of the chance to begin repairs, the lungs reveal their diseased condition—*i. e.*, they proceed to rid themselves of the accumulated impurities. Persistent in-door life would have aggravated the evil by postponing the crisis, or by turning the temporary affection into a chronic disease."

To get rid of the fresh-air superstition it is only necessary for the physician—who is sufficiently enlightened—to explain to patients the necessity of air, and to insist that the sick particularly shall have it. By doing this many sufferers will rise up and call him the true physician—the healer. G. H. C.

---

MERCURIAL DENTISTRY.—The late Dr. R. R. Gregg, of Buffalo, was the author of several articles showing the harmful effects of mercury as used by dentists for tooth-filling, for dentures, and for other purposes. Slight observation on the part of any homœopathician will show that Dr. Gregg was right in calling mercurial dentistry a "curse."

We are frequently called upon to prescribe for throat affections and other ailments which we are positive are due to amalgam fillings in the teeth, and we find that the sufferers are never entirely rid of their distressing symptoms until such fillings are removed. Not only are acute symptoms due to this cause, but, as one having a knowledge of the effects of mercury would expect, there is marked chronicity in such cases, and at times constant ailments which can only be permanently relieved by removing the cause. Dentists invariably deny that any systemic effects are due to this cause, but their denial is of no weight in presence of the symptoms.

Physicians should caution patients against the use of mercury in the teeth, and by so doing they will save much suffering and have less difficulty in curing many affections. G. H. C.

---

INFANT FOOD.—The season is again at hand when stomach and bowel troubles, particularly in infants, prevail, and then the question of the proper kind of food is appropriate. After some experience with the below mentioned food we can heartily commend it in cases needing other than the mother's milk :

"Put four tablespoonfuls of rice into three pints of water and boil half an hour ; then set aside on the back of the range to simmer during the day, water being occasionally added to maintain the original three pints. At night strain through a colander and place on ice. When cold a paste is formed. Three tablespoonfuls of this paste are added to each nursing-bottle (half pint) of milk, and fed during the next day, a fresh supply of rice-paste being under way in the meantime. If constipation be present farina may be prepared in the same way, and in the same proportion." G. H. C.

---

THE PSORA THEORY AND DR. REUTER'S OBSERVATIONS.—In our March number we adverted to Hahnemann's Psora theory, and to the observations of Dr. Reuter in connection therewith. We think the following goes to prove the truth of those observations.

Twenty years ago the late Dr. Lippe said to us: "Some time back toothache was a common complaint among patients. Now I rarely see a case of that trouble. Catarrh has taken its place, and is now more often met with than ever before."

This was before Dr. Reuter's observations were given to the profession through Grauvogl.

Turning to Dr. Reuter's list we find toothache removed four points from catarrhs. Then, using Dr. Lippe's observations, we can but conclude that the intervening affections have to a great extent disappeared. In this connection it must not be forgotten that the large majority of Dr. Lippe's patients had been under his—Hahnemannian—treatment for a number of years, and that the result was a disappearance of ailments which sequentially appear in those of a psoric taint, and an advance toward a normal condition of system.

Here is a field for observation which we trust will not be left untilled. G. H. C.

---

DR. WELLS ON INTERMITTENT FEVER.—In this number we give the first installment of a book upon "Intermittent Fever" by the venerable Dr. Wells, of Brooklyn.

The book was written years ago; but Dr. Wells could not decide to publish it and so it was laid aside. We have happily succeeded in overcoming Dr. Wells' scruples and he has placed the manuscript in our hands with permission to publish it in full.

We consider ourselves fortunate in securing this excellent work for publication, and our readers will certainly congratulate themselves upon the privileges they enjoy in THE HOMŒOPATHIC PHYSICIAN, which, in addition to all the valuable assistance it has afforded in the past to earnest seekers after the simillimum, now offers them this additional treasure.

Dr. Wells has become very feeble by reason of repeated strokes of paralysis, and is therefore no longer able to practice his profession, nor to write his strong articles in defense of the cause. But he continues to take a lively interest in everything that happens in homœopathic circles. It is our privilege frequently to see him and give him information upon the occurrences of the day. The preface of his book he dictated to us a few days since in an interview. We wrote it out and submitted it to his inspection. It is, therefore, the latest utterance of the venerable Hahnemannian. W. M. J.

